# Supplementary material for: Identifying brain network topology changes in task processes and psychiatric disorders
Source: Netw Neurosci. 2020 Mar 1;4(1):257–73. doi: 10.1162/netn_a_00122 (PMC7069064; doi:10.1162/netn_a_00122)
Supplement: Supplementary file 1 [file netn-04-257-s001.pdf]

Rezaeinia, P., Fairley, K., Pal, P., Meyer, F. G., & Carter, R. M. (2020). Supporting information for "Identifying Brain Network Topology Changes in Task Processes and Psychiatric Disorders." *Network Neuroscience*, *4*(1), 257–273. https://doi.org/10.1162/netn_a_00122

RESEARCH

Identifying Brain Network Topology Changes in Task Processes and Psychiatric Disorders: Supportive Information

Paria Rezaeinia[1], **Kim Fairley**[2,3], **Piya Pal**[1], **François G. Meyer**[4] **& R. McKell Carter**[3,5,6]

[1]Department of Electrical and Computer Engineering, University of California San Diego, San Diego, U.S.A.

[2]Department of Economics, Leiden University, Leiden, The Netherlands

[3]Institute of Cognitive Science, University of Colorado Boulder, Boulder, U.S.A.

[4]Department of Applied Mathematics, University of Colorado Boulder, Boulder, U.S.A.

[5]Department of Electrical, Computer, and Energy Engineering, University of Colorado Boulder, Boulder, U.S.A.

[6]Department of Psychology and Neuroscience, University of Colorado Boulder, Boulder, U.S.A.

Keywords: (fMRI, functional connectivity, random walk, hitting time)

SUPPORTIVE INFORMATION

Data.

We used the functional magnetic resonance imaging (fMRI) data from the LA5c Study Poldrack et al. (2016), collected by the UCLA Consortium for Neuropsychiatric Phenomics (CNP), which is funded by the NIH Roadmap Initiative. This data was obtained from the OpenfMRI database. Its accession number is ds000030. The dataset is formatted according to the Brain Imaging Data Structure K. J. Gorgolewski et al. (2016) (BIDS) standard. This study contains neuroimaging data from 290 participants. We ended up with a sample number of 255 subjects after removing subjects with missing functional measurements. In this sample, there are 119 healthy individuals (labeled as control), 49 individuals diagnosed with schizophrenia, 48 individuals diagnosed with bipolar disorder and lastly 39 individuals diagnosed with attention deficit hyperactivity disorder (ADHD). The focus of the LA5c Study is to understand memory and cognitive functional structures across patient groups. Therefore, the data set includes resting-state fMRI data as well as fMRI data collected during several different tasks. In this paper we focus on the analysis of resting-state and balloon analogue risk task (BART) fMRI data for the four specified groups. We focused on the BART because it has reliable visual input which has been shown to produce large-scale changes in sensory cortical hierarchies, which could be compared to the internally-driven resting-state network. In addition,

we develop variants of the BART and so we have expertise with the task and any findings would have local applications. We obtained the preprocessed data which is de-identified, motion corrected and coregistered to Montreal Neurological Institute (MNI) standard space K. Gorgolewski, Durnez, and Poldrack (2017). We used FSL Jenkinson, Beckmann, Behrens, Woolrich, and Smith (2012); Smith et al. (2004) to correct for motion and apply a high-pass filter to remove low-frequency noise (cut-off frequency of 100 seconds). The data also includes potential confound regressors K. Gorgolewski et al. (2017). In order to remove the effect of motion artifacts, we used the 36-parameter motion regression technique introduced in Satter et al. Satterthwaite et al. (2013), which has been shown to be most effective in decoupling modular structure from subject motion Ciric et al. (2017).

Parcellation.

The fMRI data has the following parameters; the matrix consists of 64×64 voxels for 34 slices recorded with a TR of 2 seconds. In total, there are 147 time samples for resting-state data and 267 time samples for BART data. To extract a reliable cortical network for each participant, we reduced the number of nodes by averaging over voxels within an anatomical region. We used the multi-modal parcellation developed by Glasser, et al. Glasser et al. (2016) to map the fMRI data into a more sparse decomposition framework. There are 180 regions in each hemisphere, which increase the neuroanatomical precision for studying the structural and functional organization. This parcellation is based on multiple neurobiological properties, connectivity, functional and architecture, which improves the consistency across subjects.

HITTING TIME VS. DEGREE DISTRIBUTION

To compare the ability of hitting time and degree distributions to distinguish the presence of linear components, we generated a random graph with $N = 50$ nodes in which a connection between each node randomly occurred with $p = 0.6$. We then attached a linear component of length 1 to the graph and kept increasing its length by 1 for 20 iterations (Fig. 1A). Skewness of the hitting-time distribution increases as we increase the length of linear component (Fig. 1B, blue/solid). The skewness of degree distribution remains close to constant (Fig. 1B, black/dashed). The change in other features of degree distribution such as mean or median do not reliably reflect the linear component either. Depending on the relative size of network and linear component, we might observe change in the degree distribution, but it is not consistent and is not most closely related to the presence of linear components.

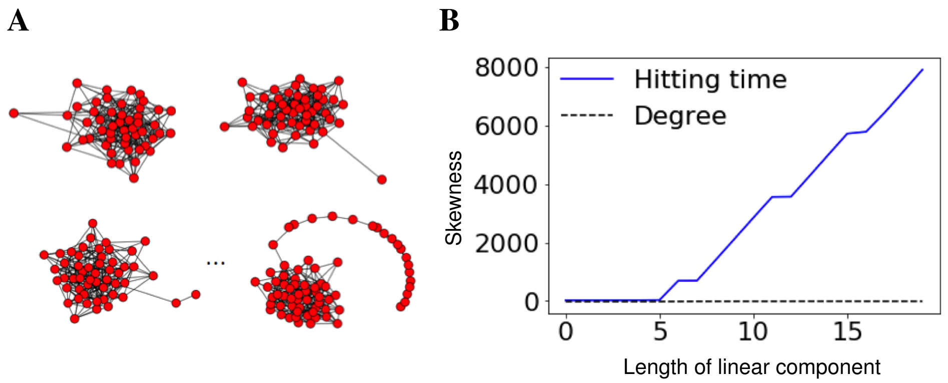

Figure 1: The hitting-time distribution of a graph becomes more skewed as the length of a linear subgraph increases. (A) We started with a random graph with 50 nodes, added a linear component of length 1 and increased the length of the linear component by 1 for 20 iterations. (B) Skewness of the hitting-time distribution increased significantly as the length of linear component increased. The degree distribution does not demonstrate a consistent relationship with linear path length.

MEAN AND MEDIAN OF HITTING-TIME DISTRIBUTION

Effect of task on mean and median of hitting-time distribution.

Aligned with the tests for skewness, we seek the effect of task on mean and median of hitting-time distribution. We ran a linear mixed effect model by adding a random effect for participant (BART and resting-state points were paired by participant). We found that mean of hitting-time distribution is significantly smaller for BART compared to rest ($\beta = -1.6, t(118) = 0.13, p < 0.001$).

Finally, a linear mixed effects model on the median of hitting times reveals a significant positive effect of BART vs. rest, ($\beta = 1.3, t(118) = 0.16, p < 0.001$), Fig. 2.

Effect of psychiatric disorders on mean and median of hitting-time distribution.

To test the effect of psychiatric disorders on the average efficiency, we run an ordinary least squares regression model with mean of hitting-time distribution as the dependent variable. The independent variables are group (coded as a dummy variable with the control group as the reference group), gender (coded as a dummy variable with the female gender acted as the reference category) and age (mean centered, linear). We found significant differences between schizophrenia versus control ($\beta = -1.113, t(252) = 0.255, p < 0.001$), bipolar versus control

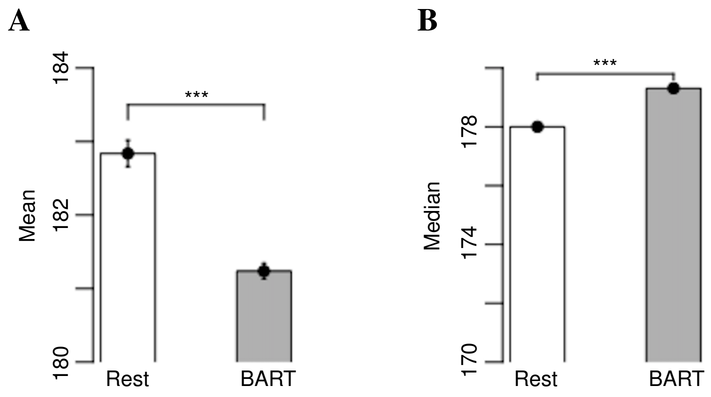

Figure 2: The distribution of (A) mean and (B) median vs. task for control subjects. p-values significance codes: $. = 0.1, * =< 0.05, ** =< 0.01, *** =< 0.001$.

$(\beta = -1.064, t(252) = 0.252, p < 0.001)$ and ADHD patients versus control $(\beta = -0.835, t(252) = 0.266, p = 0.002)$. The qualitative results are similar to skewness, but the coefficients are approximately one quarter the magnitude of those in the model explaining skewness. Finally, if we run a similar model with the median of the resting-state hitting-time distribution as the dependent variable, we only find a trend toward significance between schizophrenia and control in the opposite direction $(\beta = 0.473, t(252) = 0.244, p = 0.054)$. Hierarchical sensory processing streams identified above as those with the longest hitting times show the largest changes between control and diagnosed groups, Fig. (3).

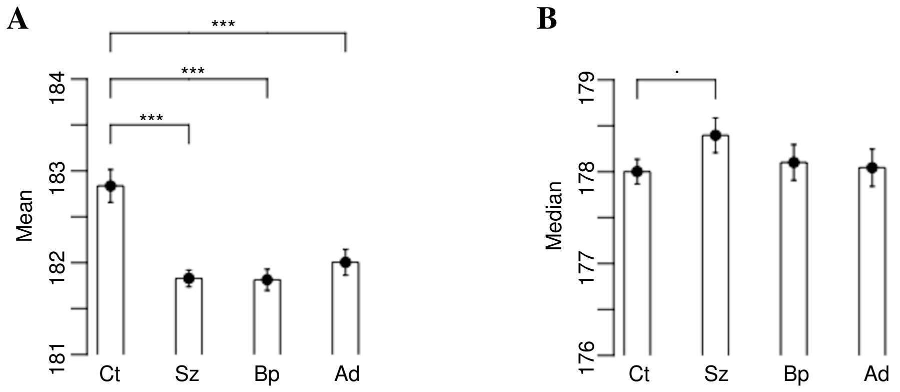

Figure 3: Mean of hitting-time distribution is significantly different across patient groups. Distribution (A) mean and (B) median of the hitting-time distribution in patient and control groups during resting-state scans. p-values significance codes: $. = 0.1, * =< 0.05, ** =< 0.01, *** =< 0.001$.

REFERENCES

Ciric, R., Wolf, D. H., Power, J. D., Roalf, D. R., Baum, G. L., Ruparel, K., … Satterthwaite, T. D. (2017). Benchmarking of participant-level confound regression strategies for the control of motion artifact in studies of functional connectivity. *Neuroimage*, *154*, 174–187. doi: 10.1016/j.neuroimage.2017.03.020

Glasser, M. F., Coalson, T. S., Robinson, E. C., Hacker, C. D., Harwell, J., Yacoub, E., … Van Essen, D. C. (2016). A multi-modal parcellation of human cerebral cortex. *Nature*, *536*, 171 EP -. Retrieved from http://dx.doi.org/10.1038/nature18933

Gorgolewski, K., Durnez, J., & Poldrack, R. (2017). Preprocessed consortium for neuropsychiatric phenomics dataset [version 1; referees: 2 approved with reservations]. *F1000Research*, *6*(1262). doi: 10.12688/f1000research.11964.1

Gorgolewski, K. J., Auer, T., Calhoun, V. D., Craddock, R. C., Das, S., Duff, E. P., … Poldrack, R. A. (2016). The brain imaging data structure, a format for organizing and describing outputs of neuroimaging experiments. *Sci Data*, *3*, 160044. doi: 10.1038/sdata.2016.44

Jenkinson, M., Beckmann, C. F., Behrens, T. E. J., Woolrich, M. W., & Smith, S. M. (2012). Fsl. *NeuroImage*, *62*(2), 782–790.

Poldrack, R., Congdon, E., Triplett, W., Gorgolewski, K., Karlsgodt, K., Mumford, J., … Bilder, R. (2016). A phenome-wide examination of neural and cognitive function. *bioRxiv*. Retrieved from http://biorxiv.org/content/early/2016/06/19/059733 doi: 10.1101/059733

Satterthwaite, T. D., Elliott, M. A., Gerraty, R. T., Ruparel, K., Loughead, J., Calkins, M. E., … Wolf, D. H. (2013). An improved framework for confound regression and filtering for control of motion artifact in the preprocessing of resting-state functional connectivity data. *Neuroimage*, *64*, 240–256. doi: 10.1016/j.neuroimage.2012.08.052

Smith, S. M., Jenkinson, M., Woolrich, M. W., Beckmann, C. F., Behrens, T. E. J., Johansen-Berg, H., … Matthews, P. M. (2004). Advances in functional and structural mr image analysis and implementation as fsl. *Neuroimage*, *23 Suppl 1*, S208-19. doi: 10.1016/j.neuroimage.2004.07.051
